# Crystal structure of (*E*)-diethyl 2-[(1-phenyl­sulfonyl-1*H*-indol-3-yl)methyl­idene]succinate

**DOI:** 10.1107/S2056989015023075

**Published:** 2015-12-09

**Authors:** M. Umadevi, Potharaju Raju, R. Yamuna, Arasambattu K. Mohanakrishnan, G. Chakkaravarthi

**Affiliations:** a Research and Development Centre, Bharathiar University, Coimbatore 641 046, India; b Department of Chemistry, Pallavan College of Engineering, Kanchipuram 631 502, India; c Department of Organic Chemistry, University of Madras, Guindy Campus, Chennai 600 025, India; d Department of Sciences, Chemistry and Materials Research Lab, Amrita Vishwa Vidyapeetham University, Ettimadai, Coimbatore 641 112, India; e Department of Physics, CPCL Polytechnic College, Chennai 600 068, India

**Keywords:** crystal structure, indole derivative, hydrogen bonding

## Abstract

In the title compound, C_23_H_23_NO_6_S, the phenyl ring is perpendicular [dihedral angle = 89.34 (9)°] to the indole ring system. In the mol­ecule, the eth­oxy groups are each disordered over two sets of sites with occupancy ratios of 0.671 (6):0.329 (6) and 0.75 (3):0.25 (3). The mol­ecular conformation is consolidated by a weak C—H⋯O interaction, which generates an *S*(6) graph–set motif. The packing of the mol­ecules in the crystal structure features weak C—H⋯π inter­actions.

## Related literature   

For the biological activity of indole derivatives, see: Andreani *et al.* (2001 ▸); Kolocouris *et al.* (1994 ▸). For the structures of closely related compounds, see: Chakkaravarthi *et al.* (2007 ▸, 2008 ▸).
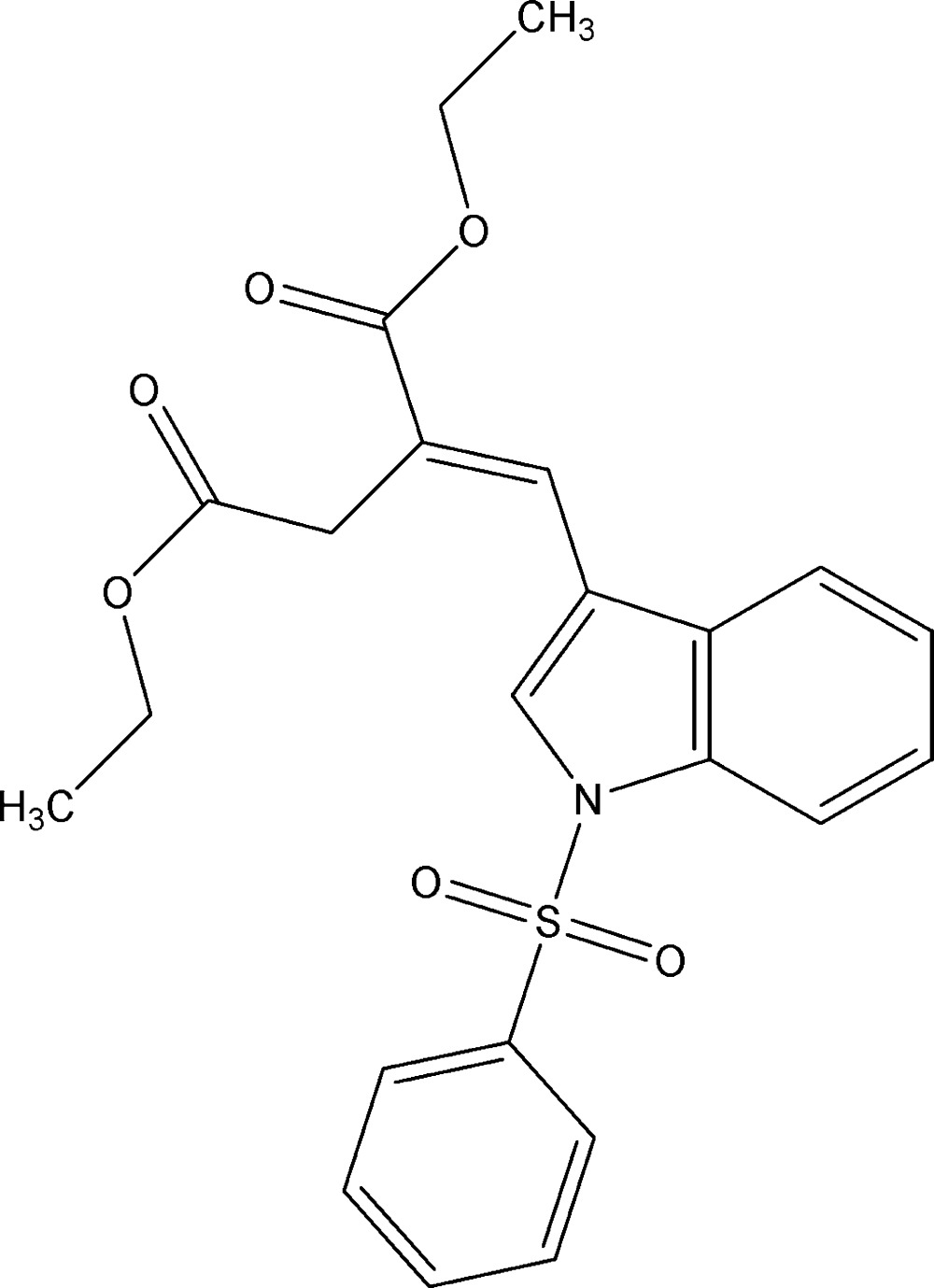


## Experimental   

### Crystal data  

C_23_H_23_NO_6_S
*M* *_r_* = 441.48
Monoclinic, 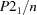

*a* = 8.3458 (8) Å
*b* = 24.657 (2) Å
*c* = 10.9448 (9) Å
β = 91.121 (3)°
*V* = 2251.8 (3) Å^3^
*Z* = 4
Mo *K*α radiation
μ = 0.18 mm^−1^
*T* = 295 K
0.30 × 0.24 × 0.20 mm

### Data collection  

Bruker Kappa APEXII CCD diffractometer
Absorption correction: multi-scan (*SADABS*; Sheldrick, 1996 ▸) *T*_min_ = 0.947, *T*_max_ = 0.965
31225 measured reflections
4631 independent reflections
2889 reflections with *I* > 2σ(*I*)
*R*_int_ = 0.045

### Refinement  

*R*[*F* ^2^ > 2σ(*F* ^2^)] = 0.049
*wR*(*F* ^2^) = 0.139
*S* = 1.03
4631 reflections
322 parameters
5 restraints
H-atom parameters constrained
Δρ_max_ = 0.31 e Å^−3^
Δρ_min_ = −0.35 e Å^−3^

### 

Data collection: *APEX2* (Bruker, 2004 ▸); cell refinement: *SAINT* (Bruker, 2004 ▸); data reduction: *SAINT*; program(s) used to solve structure: *SHELXS97* (Sheldrick, 2008 ▸); program(s) used to refine structure: *SHELXL97* (Sheldrick, 2008 ▸); molecular graphics: *PLATON* (Spek, 2009 ▸); software used to prepare material for publication: *SHELXL97*.

## Supplementary Material

Crystal structure: contains datablock(s) global, I. DOI: 10.1107/S2056989015023075/rk2433sup1.cif

Structure factors: contains datablock(s) I. DOI: 10.1107/S2056989015023075/rk2433Isup2.hkl

Supporting information file. DOI: 10.1107/S2056989015023075/rk2433Isup3.cml

**Fig. 1.**
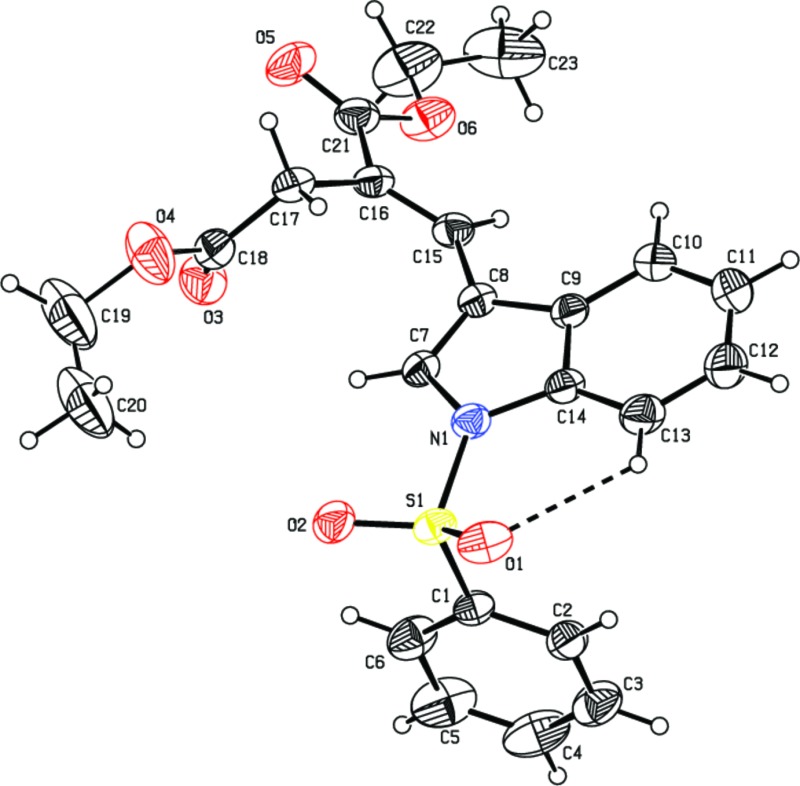
The molecular structure of title compound, with atom labels. Displacement ellipsoids are drawn at 30% probability level. The H atoms are presented as a small spheres of arbitrary radius. The minor components of the disordered ethyl groups are omitted for clarity.

. DOI: 10.1107/S2056989015023075/rk2433fig1.tif
The mol­ecular structure of title compound, with atom labels. Displacement ellipsoids are drawn at 30% probability level. The H atoms are presented as a small spheres of arbitrary radius. The minor components of the disordered ethyl groups are omitted for clarity.

CCDC reference: 1439879

Additional supporting information:  crystallographic information; 3D view; checkCIF report

## Figures and Tables

**Table 1:** Hydrogen-bond geometry (Å, °) *Cg*2 is the centroid of C1–C6 ring.

*D*—H⋯*A*	*D*—H	H⋯*A*	*D*⋯*A*	*D*—H⋯*A*
C13—H13⋯O1	0.93	2.44	3.012 (4)	120
C12—H12⋯*Cg*2^i^	0.93	2.80	3.561 (4)	140

Symmetry code: (i) 
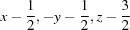
.

## supplementary crystallographic information

### S1. Structural commentary

Indole derivatives exhibit anti­tumour (Andreani *et al.*, 2001) and anti­viral (Kolocouris *et al.*, 1994) activities. The molecular structure of the title compound is illustrated in (Fig. 1). The geometric parameters of the title molecule agree well with the reported similar structures (Chakkaravarthi *et al.* 2007, 2008).

The phenyl ring (C1–C6) is perpendicular [dihedral angle of 89.34 (9)°] to indole ring (N1/C7–C14) system in the molecule. In the terminal site, the eth­oxy group is disordered over two positions with site occupancies of 0.671 (6) for major component (O4/C19/C20) and 0.329 (6) for minor component (O4A/C19A/C20A). The other eth­oxy group is also disordered over two positions with site occupancies of 0.75 (3) for major component (O6/C23/C24) and 0.25 (3) for minor component (O6A/C23A/C24A). The torsion angles O1—S1—N1—C14 and O2—S1—N1—C7 [39.9 (2)° and −32.8 (2)°, respectively] indicate the *syn*–conformation of the sulfonyl moiety. The molecular structure is stabilized by weak non–classical C—H···O hydrogen bond which generates S(6) graph–set (Table 1 & Fig. 1) motif. The crystal structure is influenced by weak C—H···π (Table 1) inter­actions in a three–dimensional network.

### S2. Synthesis and crystallization

To a solution of 1–(phenyl­sulfonyl)–1*H*–indole–3–carboxaldehyde (0.5 g, 1.84 mmol) and phospho­rous ylide (C_26_H_27_O_4_P) (0.96 g, 2.21 mmol) [prepared from (carbeth­oxy­methyl­ene)tri­phenyl­phospho­rane and ethyl bromo­acetate] in dry toluene (10 ml) was refluxed for 24 h. Then the solvent was removed under reduced pressure. The solid obtained was recrystallized from methanol (3 ml) to afford the title compound as a colourless crystal suitable for *X*–Ray diffraction analysis.

### S3. Refinement

The H atoms were positioned geometrically and refined using riding model, with C—H = 0.93 Å and *U*_iso_(H) = 1.2*U*_eq_(C) for aromatic H, C—H = 0.97 Å and *U*_iso_(H) = 1.2*U*_eq_(C) for methyl­ene H and C—H = 0.96 Å and *U*_iso_(H) = 1.5*U*_eq_(C) for methyl H.

The reflections [1 2 2], [0 2 0] and [0 1 1] were omitted during refinement which were owing poor agreement. The bond distances (C19—C20), (C19A—C20A), (C22—C23) and (C22A—C23A) were restraint to 1.54 (1) Å. The anisotropic displacement parameters of terminal site disordered atoms were equalized for the major and minor components with *EADP* instruction for C20 & C20A and C19 & C19A. The anisotropic displacement parameters in the direction of S1 and O2 were restraint within 0.001 using *DELU* instruction in *SHELXL* refinement.

### Crystal data

**Table:**

C_23_H_23_NO_6_S	*F*(000) = 928
*M**_r_* = 441.48	*D*_x_ = 1.302 Mg m^−^^3^
Monoclinic, *P*2_1_/*n*	Mo *K*α radiation, λ = 0.71073 Å
Hall symbol: -P 2yn	Cell parameters from 6504 reflections
*a* = 8.3458 (8) Å	θ = 2.5–23.4°
*b* = 24.657 (2) Å	µ = 0.18 mm^−^^1^
*c* = 10.9448 (9) Å	*T* = 295 K
β = 91.121 (3)°	Block, colourless
*V* = 2251.8 (3) Å^3^	0.30 × 0.24 × 0.20 mm
*Z* = 4	

### Data collection

**Table:**

Bruker Kappa APEXII CCD diffractometer	4631 independent reflections
Radiation source: fine–focus sealed tube	2889 reflections with *I* > 2σ(*I*)
Graphite monochromator	*R*_int_ = 0.045
ω and φ scan	θ_max_ = 26.5°, θ_min_ = 2.5°
Absorption correction: multi-scan (*SADABS*; Sheldrick, 1996)	*h* = −10→10
*T*_min_ = 0.947, *T*_max_ = 0.965	*k* = −30→30
31225 measured reflections	*l* = −13→13

### Refinement

**Table:**

Refinement on *F*^2^	Primary atom site location: structure-invariant direct methods
Least-squares matrix: full	Secondary atom site location: difference Fourier map
*R*[*F*^2^ > 2σ(*F*^2^)] = 0.049	Hydrogen site location: inferred from neighbouring sites
*wR*(*F*^2^) = 0.139	H-atom parameters constrained
*S* = 1.03	*w* = 1/[σ^2^(*F*_o_^2^) + (0.0555*P*)^2^ + 0.8923*P*] where *P* = (*F*_o_^2^ + 2*F*_c_^2^)/3
4631 reflections	(Δ/σ)_max_ < 0.001
322 parameters	Δρ_max_ = 0.31 e Å^−^^3^
5 restraints	Δρ_min_ = −0.35 e Å^−^^3^

### Special details

**Table:**

Geometry. All s.u.'s (except the s.u. in the dihedral angle between two l.s. planes) are estimated using the full covariance matrix. The cell s.u.'s are taken into account individually in the estimation of s.u.'s in distances, angles and torsion angles; correlations between s.u.'s in cell parameters are only used when they are defined by crystal symmetry. An approximate (isotropic) treatment of cell s.u.'s is used for estimating s.u.'s involving l.s. planes.
Refinement. Refinement of **F**^2^ against ALL reflections. The weighted **R**–factor w**R** and goodness of fit **S** are based on **F**^2^, conventional **R**–factors **R** are based on **F**, with **F** set to zero for negative **F**^2^. The threshold expression of **F**^2^ > 2σ(**F**^2^) is used only for calculating **R**–factors(gt) *etc*. and is not relevant to the choice of reflections for refinement. **R**–factors based on **F**^2^ are statistically about twice as large as those based on **F**, and **R**–factors based on ALL data will be even larger.

### Fractional atomic coordinates and isotropic or equivalent isotropic displacement parameters (Å^2^)

**Table:**

	*x*	*y*	*z*	*U*_iso_*/*U*_eq_	Occ. (<1)
C1	0.6466 (3)	0.30006 (9)	0.3924 (2)	0.0459 (6)	
C2	0.6500 (3)	0.26226 (10)	0.2988 (2)	0.0567 (7)	
H2	0.7465	0.2522	0.2641	0.068*	
C3	0.5073 (4)	0.23988 (12)	0.2582 (3)	0.0763 (9)	
H3	0.5069	0.2145	0.1953	0.092*	
C4	0.3674 (5)	0.25465 (15)	0.3095 (4)	0.0955 (12)	
H4	0.2717	0.2396	0.2808	0.115*	
C5	0.3651 (4)	0.29161 (15)	0.4035 (4)	0.0997 (13)	
H5	0.2686	0.3007	0.4393	0.120*	
C6	0.5061 (4)	0.31516 (11)	0.4447 (3)	0.0715 (8)	
H6	0.5056	0.3408	0.5069	0.086*	
C7	0.7896 (3)	0.43043 (9)	0.3542 (2)	0.0478 (6)	
H7	0.7400	0.4439	0.4232	0.057*	
C8	0.8039 (3)	0.45752 (9)	0.2475 (2)	0.0454 (6)	
C9	0.8822 (3)	0.42149 (9)	0.1642 (2)	0.0485 (6)	
C10	0.9268 (4)	0.42694 (12)	0.0431 (3)	0.0663 (8)	
H10	0.9071	0.4590	0.0006	0.080*	
C11	1.0001 (4)	0.38407 (14)	−0.0123 (3)	0.0844 (10)	
H11	1.0287	0.3869	−0.0937	0.101*	
C12	1.0325 (4)	0.33661 (13)	0.0507 (3)	0.0806 (10)	
H12	1.0828	0.3082	0.0107	0.097*	
C13	0.9924 (3)	0.33027 (11)	0.1709 (3)	0.0662 (8)	
H13	1.0155	0.2985	0.2134	0.079*	
C14	0.9159 (3)	0.37344 (9)	0.2258 (2)	0.0485 (6)	
C15	0.7460 (3)	0.51183 (9)	0.2172 (2)	0.0505 (6)	
H15	0.7002	0.5161	0.1396	0.061*	
C16	0.7508 (3)	0.55562 (9)	0.2867 (2)	0.0477 (6)	
C17	0.8300 (3)	0.55776 (10)	0.4103 (2)	0.0528 (7)	
H17A	0.9083	0.5288	0.4167	0.063*	
H17B	0.8869	0.5919	0.4188	0.063*	
C18	0.7145 (4)	0.55251 (11)	0.5119 (3)	0.0588 (7)	
O4	0.7948 (8)	0.5510 (3)	0.6172 (6)	0.104 (2)	0.671 (6)
C19	0.6975 (11)	0.5455 (4)	0.7255 (6)	0.152 (3)	0.671 (6)
H19A	0.5860	0.5536	0.7065	0.182*	0.671 (6)
H19B	0.7350	0.5698	0.7897	0.182*	0.671 (6)
C20	0.7170 (10)	0.4872 (4)	0.7640 (6)	0.152 (3)	0.671 (6)
H20A	0.6600	0.4642	0.7073	0.228*	0.671 (6)
H20B	0.6750	0.4824	0.8443	0.228*	0.671 (6)
H20C	0.8287	0.4778	0.7649	0.228*	0.671 (6)
O4A	0.7807 (15)	0.5214 (4)	0.6047 (10)	0.066 (2)	0.329 (6)
C19A	0.677 (3)	0.5038 (8)	0.7017 (13)	0.152 (3)	0.329 (6)
H19C	0.7058	0.4669	0.7233	0.182*	0.329 (6)
H19D	0.5678	0.5030	0.6694	0.182*	0.329 (6)
C20A	0.678 (2)	0.5367 (8)	0.8154 (11)	0.152 (3)	0.329 (6)
H20D	0.7847	0.5369	0.8506	0.228*	0.329 (6)
H20E	0.6055	0.5212	0.8725	0.228*	0.329 (6)
H20F	0.6463	0.5731	0.7965	0.228*	0.329 (6)
C21	0.6780 (4)	0.60772 (11)	0.2456 (3)	0.0597 (7)	
O6	0.605 (2)	0.6030 (5)	0.1389 (16)	0.076 (3)	0.75 (3)
C22	0.501 (2)	0.6514 (6)	0.1022 (11)	0.111 (4)	0.75 (3)
H22A	0.3980	0.6502	0.1414	0.133*	0.75 (3)
H22B	0.5540	0.6854	0.1226	0.133*	0.75 (3)
C23	0.4834 (17)	0.6446 (6)	−0.0316 (10)	0.129 (5)	0.75 (3)
H23A	0.5874	0.6412	−0.0668	0.193*	0.75 (3)
H23B	0.4293	0.6755	−0.0659	0.193*	0.75 (3)
H23C	0.4220	0.6125	−0.0490	0.193*	0.75 (3)
O6A	0.569 (7)	0.6022 (18)	0.153 (5)	0.096 (12)	0.25 (3)
C22A	0.567 (3)	0.6554 (16)	0.084 (4)	0.077 (7)	0.25 (3)
H22C	0.6598	0.6596	0.0334	0.093*	0.25 (3)
H22D	0.5576	0.6863	0.1384	0.093*	0.25 (3)
C23A	0.415 (4)	0.6461 (18)	0.009 (5)	0.138 (15)	0.25 (3)
H23D	0.4365	0.6217	−0.0570	0.207*	0.25 (3)
H23E	0.3773	0.6801	−0.0238	0.207*	0.25 (3)
H23F	0.3339	0.6308	0.0597	0.207*	0.25 (3)
N1	0.8600 (2)	0.37943 (7)	0.34558 (19)	0.0484 (5)	
O1	0.9526 (2)	0.29189 (7)	0.42972 (19)	0.0672 (6)	
O2	0.8019 (2)	0.35559 (7)	0.55794 (16)	0.0678 (5)	
O3	0.5734 (3)	0.55723 (9)	0.5029 (2)	0.0778 (6)	
O5	0.6935 (3)	0.64938 (7)	0.2993 (2)	0.0852 (7)	
S1	0.82591 (8)	0.32957 (2)	0.44398 (6)	0.0503 (2)	

### Atomic displacement parameters (Å^2^)

**Table:**

	*U*^11^	*U*^22^	*U*^33^	*U*^12^	*U*^13^	*U*^23^
C1	0.0451 (14)	0.0345 (12)	0.0580 (15)	0.0042 (11)	−0.0034 (12)	0.0042 (11)
C2	0.0622 (18)	0.0516 (15)	0.0561 (16)	−0.0040 (13)	−0.0039 (13)	0.0003 (12)
C3	0.083 (2)	0.0685 (19)	0.077 (2)	−0.0151 (18)	−0.0250 (18)	−0.0036 (16)
C4	0.062 (2)	0.073 (2)	0.151 (4)	−0.0129 (18)	−0.035 (2)	0.005 (2)
C5	0.049 (2)	0.079 (2)	0.171 (4)	0.0067 (18)	0.009 (2)	−0.003 (3)
C6	0.0562 (19)	0.0530 (16)	0.105 (2)	0.0061 (14)	0.0061 (17)	−0.0103 (16)
C7	0.0529 (15)	0.0371 (12)	0.0533 (15)	0.0003 (11)	0.0010 (12)	−0.0034 (11)
C8	0.0440 (14)	0.0376 (12)	0.0546 (15)	−0.0014 (11)	0.0003 (11)	−0.0006 (11)
C9	0.0478 (15)	0.0429 (13)	0.0549 (15)	−0.0076 (11)	0.0051 (12)	−0.0045 (11)
C10	0.078 (2)	0.0557 (16)	0.0657 (19)	−0.0134 (15)	0.0154 (16)	−0.0019 (14)
C11	0.100 (3)	0.079 (2)	0.076 (2)	−0.016 (2)	0.0355 (19)	−0.0140 (18)
C12	0.086 (2)	0.064 (2)	0.093 (3)	−0.0013 (17)	0.036 (2)	−0.0192 (18)
C13	0.0634 (19)	0.0469 (15)	0.089 (2)	0.0050 (14)	0.0201 (16)	−0.0047 (14)
C14	0.0420 (14)	0.0398 (13)	0.0640 (17)	−0.0027 (11)	0.0070 (12)	−0.0047 (11)
C15	0.0556 (16)	0.0413 (13)	0.0543 (15)	−0.0008 (11)	−0.0018 (12)	0.0085 (11)
C16	0.0479 (14)	0.0352 (12)	0.0601 (16)	−0.0023 (11)	0.0053 (12)	0.0058 (11)
C17	0.0507 (15)	0.0410 (13)	0.0667 (17)	−0.0065 (11)	−0.0003 (13)	−0.0009 (11)
C18	0.0604 (19)	0.0555 (16)	0.0604 (18)	−0.0053 (14)	0.0010 (15)	−0.0051 (13)
O4	0.077 (3)	0.180 (7)	0.056 (3)	−0.019 (4)	−0.006 (2)	−0.001 (4)
C19	0.125 (4)	0.269 (9)	0.063 (3)	−0.051 (6)	0.014 (3)	0.008 (4)
C20	0.125 (4)	0.269 (9)	0.063 (3)	−0.051 (6)	0.014 (3)	0.008 (4)
O4A	0.068 (5)	0.082 (6)	0.048 (4)	−0.018 (5)	−0.002 (3)	−0.003 (4)
C19A	0.125 (4)	0.269 (9)	0.063 (3)	−0.051 (6)	0.014 (3)	0.008 (4)
C20A	0.125 (4)	0.269 (9)	0.063 (3)	−0.051 (6)	0.014 (3)	0.008 (4)
C21	0.0659 (19)	0.0443 (15)	0.0691 (19)	0.0003 (13)	0.0066 (16)	0.0096 (14)
O6	0.086 (5)	0.047 (4)	0.094 (5)	0.010 (4)	−0.018 (3)	0.017 (4)
C22	0.118 (10)	0.065 (4)	0.148 (9)	0.027 (8)	−0.043 (8)	0.031 (5)
C23	0.097 (7)	0.127 (7)	0.160 (9)	−0.017 (7)	−0.056 (7)	0.073 (7)
O6A	0.11 (3)	0.078 (18)	0.095 (17)	0.055 (14)	−0.047 (17)	−0.023 (13)
C22A	0.054 (11)	0.060 (12)	0.119 (15)	0.028 (11)	0.013 (12)	0.021 (9)
C23A	0.071 (17)	0.107 (18)	0.23 (4)	0.021 (18)	−0.03 (2)	0.02 (2)
N1	0.0516 (12)	0.0369 (10)	0.0567 (13)	0.0023 (9)	0.0038 (10)	0.0013 (9)
O1	0.0516 (11)	0.0501 (10)	0.0993 (15)	0.0089 (9)	−0.0161 (10)	0.0131 (10)
O2	0.0949 (15)	0.0554 (11)	0.0524 (9)	−0.0092 (10)	−0.0127 (10)	−0.0002 (8)
O3	0.0607 (14)	0.0908 (15)	0.0824 (15)	0.0018 (12)	0.0100 (11)	−0.0054 (11)
O5	0.125 (2)	0.0359 (10)	0.0949 (16)	0.0041 (11)	0.0001 (14)	−0.0016 (10)
S1	0.0524 (4)	0.0392 (3)	0.0587 (4)	−0.0003 (3)	−0.0119 (3)	0.0059 (3)

### Geometric parameters (Å, º)

**Table:**

C1—C6	1.366 (4)	C18—O4	1.323 (7)
C1—C2	1.386 (3)	C18—O4A	1.380 (12)
C1—S1	1.748 (2)	O4—C19	1.456 (10)
C2—C3	1.379 (4)	C19—C20	1.507 (8)
C2—H2	0.9300	C19—H19A	0.9700
C3—C4	1.355 (5)	C19—H19B	0.9700
C3—H3	0.9300	C20—H20A	0.9600
C4—C5	1.375 (5)	C20—H20B	0.9600
C4—H4	0.9300	C20—H20C	0.9600
C5—C6	1.380 (4)	O4A—C19A	1.45 (2)
C5—H5	0.9300	C19A—C20A	1.485 (10)
C6—H6	0.9300	C19A—H19C	0.9700
C7—C8	1.352 (3)	C19A—H19D	0.9700
C7—N1	1.392 (3)	C20A—H20D	0.9600
C7—H7	0.9300	C20A—H20E	0.9600
C8—C9	1.439 (3)	C20A—H20F	0.9600
C8—C15	1.460 (3)	C21—O5	1.189 (3)
C9—C14	1.389 (3)	C21—O6	1.313 (17)
C9—C10	1.391 (4)	C21—O6A	1.36 (5)
C10—C11	1.369 (4)	O6—C22	1.52 (2)
C10—H10	0.9300	C22—C23	1.479 (8)
C11—C12	1.382 (5)	C22—H22A	0.9700
C11—H11	0.9300	C22—H22B	0.9700
C12—C13	1.372 (4)	C23—H23A	0.9600
C12—H12	0.9300	C23—H23B	0.9600
C13—C14	1.385 (3)	C23—H23C	0.9600
C13—H13	0.9300	O6A—C22A	1.51 (6)
C14—N1	1.408 (3)	C22A—C23A	1.516 (10)
C15—C16	1.321 (3)	C22A—H22C	0.9700
C15—H15	0.9300	C22A—H22D	0.9700
C16—C21	1.487 (3)	C23A—H23D	0.9600
C16—C17	1.494 (3)	C23A—H23E	0.9600
C17—C18	1.492 (4)	C23A—H23F	0.9600
C17—H17A	0.9700	N1—S1	1.663 (2)
C17—H17B	0.9700	O1—S1	1.4185 (18)
C18—O3	1.185 (3)	O2—S1	1.4202 (19)
			
C6—C1—C2	121.6 (3)	O4—C18—C17	109.2 (4)
C6—C1—S1	119.2 (2)	O4A—C18—C17	110.0 (6)
C2—C1—S1	119.2 (2)	C18—O4—C19	115.5 (6)
C3—C2—C1	118.5 (3)	O4—C19—C20	104.9 (7)
C3—C2—H2	120.8	O4—C19—H19A	110.8
C1—C2—H2	120.8	C20—C19—H19A	110.8
C4—C3—C2	120.3 (3)	O4—C19—H19B	110.8
C4—C3—H3	119.8	C20—C19—H19B	110.8
C2—C3—H3	119.8	H19A—C19—H19B	108.8
C3—C4—C5	120.9 (3)	C18—O4A—C19A	117.9 (13)
C3—C4—H4	119.5	O4A—C19A—C20A	117.0 (14)
C5—C4—H4	119.5	O4A—C19A—H19C	108.1
C4—C5—C6	119.9 (3)	C20A—C19A—H19C	108.1
C4—C5—H5	120.1	O4A—C19A—H19D	108.1
C6—C5—H5	120.1	C20A—C19A—H19D	108.1
C1—C6—C5	118.8 (3)	H19C—C19A—H19D	107.3
C1—C6—H6	120.6	C19A—C20A—H20D	109.5
C5—C6—H6	120.6	C19A—C20A—H20E	109.5
C8—C7—N1	110.1 (2)	H20D—C20A—H20E	109.5
C8—C7—H7	125.0	C19A—C20A—H20F	109.5
N1—C7—H7	125.0	H20D—C20A—H20F	109.5
C7—C8—C9	106.9 (2)	H20E—C20A—H20F	109.5
C7—C8—C15	128.0 (2)	O5—C21—O6	124.1 (7)
C9—C8—C15	125.1 (2)	O5—C21—O6A	121 (2)
C14—C9—C10	119.3 (2)	O5—C21—C16	123.9 (3)
C14—C9—C8	108.0 (2)	O6—C21—C16	111.9 (7)
C10—C9—C8	132.7 (2)	O6A—C21—C16	114 (2)
C11—C10—C9	118.5 (3)	C21—O6—C22	114.6 (13)
C11—C10—H10	120.7	C23—C22—O6	102.5 (13)
C9—C10—H10	120.7	C23—C22—H22A	111.2
C10—C11—C12	121.2 (3)	O6—C22—H22A	111.3
C10—C11—H11	119.4	C23—C22—H22B	111.2
C12—C11—H11	119.4	O6—C22—H22B	111.3
C13—C12—C11	121.7 (3)	H22A—C22—H22B	109.2
C13—C12—H12	119.2	C21—O6A—C22A	107 (3)
C11—C12—H12	119.2	O6A—C22A—C23A	98 (4)
C12—C13—C14	116.9 (3)	O6A—C22A—H22C	112.3
C12—C13—H13	121.6	C23A—C22A—H22C	112.1
C14—C13—H13	121.6	O6A—C22A—H22D	111.9
C13—C14—C9	122.4 (3)	C23A—C22A—H22D	112.2
C13—C14—N1	130.5 (2)	H22C—C22A—H22D	109.8
C9—C14—N1	107.1 (2)	C22A—C23A—H23D	109.5
C16—C15—C8	127.8 (2)	C22A—C23A—H23E	109.5
C16—C15—H15	116.1	H23D—C23A—H23E	109.5
C8—C15—H15	116.1	C22A—C23A—H23F	109.5
C15—C16—C21	121.6 (2)	H23D—C23A—H23F	109.5
C15—C16—C17	123.9 (2)	H23E—C23A—H23F	109.5
C21—C16—C17	114.5 (2)	C7—N1—C14	107.80 (19)
C18—C17—C16	113.0 (2)	C7—N1—S1	123.13 (18)
C18—C17—H17A	109.0	C14—N1—S1	126.13 (16)
C16—C17—H17A	109.0	O1—S1—O2	120.72 (12)
C18—C17—H17B	109.0	O1—S1—N1	106.02 (11)
C16—C17—H17B	109.0	O2—S1—N1	105.29 (10)
H17A—C17—H17B	107.8	O1—S1—C1	109.09 (11)
O3—C18—O4	124.1 (4)	O2—S1—C1	109.70 (13)
O3—C18—O4A	119.9 (6)	N1—S1—C1	104.75 (11)
O3—C18—C17	125.8 (3)		
			
C6—C1—C2—C3	0.2 (4)	C18—O4—C19—C20	104.0 (8)
S1—C1—C2—C3	−179.2 (2)	O3—C18—O4A—C19A	−11.3 (11)
C1—C2—C3—C4	−0.2 (4)	O4—C18—O4A—C19A	96.2 (17)
C2—C3—C4—C5	−0.7 (5)	C17—C18—O4A—C19A	−169.3 (9)
C3—C4—C5—C6	1.6 (6)	C18—O4A—C19A—C20A	−97.2 (18)
C2—C1—C6—C5	0.6 (4)	C15—C16—C21—O5	−172.0 (3)
S1—C1—C6—C5	−179.9 (3)	C17—C16—C21—O5	7.1 (4)
C4—C5—C6—C1	−1.5 (5)	C15—C16—C21—O6	3.4 (9)
N1—C7—C8—C9	2.2 (3)	C17—C16—C21—O6	−177.5 (8)
N1—C7—C8—C15	179.5 (2)	C15—C16—C21—O6A	19 (3)
C7—C8—C9—C14	−1.2 (3)	C17—C16—C21—O6A	−162 (3)
C15—C8—C9—C14	−178.7 (2)	O5—C21—O6—C22	−15.6 (18)
C7—C8—C9—C10	179.4 (3)	O6A—C21—O6—C22	68 (10)
C15—C8—C9—C10	1.9 (4)	C16—C21—O6—C22	169.0 (11)
C14—C9—C10—C11	1.2 (4)	C21—O6—C22—C23	160.5 (12)
C8—C9—C10—C11	−179.5 (3)	O5—C21—O6A—C22A	37 (5)
C9—C10—C11—C12	−1.2 (5)	O6—C21—O6A—C22A	−68 (9)
C10—C11—C12—C13	0.2 (5)	C16—C21—O6A—C22A	−154 (3)
C11—C12—C13—C14	0.8 (5)	C21—O6A—C22A—C23A	−167 (3)
C12—C13—C14—C9	−0.8 (4)	C8—C7—N1—C14	−2.3 (3)
C12—C13—C14—N1	179.9 (3)	C8—C7—N1—S1	−163.95 (17)
C10—C9—C14—C13	−0.1 (4)	C13—C14—N1—C7	−179.1 (3)
C8—C9—C14—C13	−179.6 (2)	C9—C14—N1—C7	1.5 (3)
C10—C9—C14—N1	179.3 (2)	C13—C14—N1—S1	−18.2 (4)
C8—C9—C14—N1	−0.2 (3)	C9—C14—N1—S1	162.42 (17)
C7—C8—C15—C16	41.3 (4)	C7—N1—S1—O1	−161.85 (19)
C9—C8—C15—C16	−141.8 (3)	C14—N1—S1—O1	39.9 (2)
C8—C15—C16—C21	−176.4 (2)	C7—N1—S1—O2	−32.8 (2)
C8—C15—C16—C17	4.6 (4)	C14—N1—S1—O2	169.0 (2)
C15—C16—C17—C18	−99.6 (3)	C7—N1—S1—C1	82.8 (2)
C21—C16—C17—C18	81.3 (3)	C14—N1—S1—C1	−75.4 (2)
C16—C17—C18—O3	−15.0 (4)	C6—C1—S1—O1	149.7 (2)
C16—C17—C18—O4	175.7 (4)	C2—C1—S1—O1	−30.8 (2)
C16—C17—C18—O4A	141.5 (4)	C6—C1—S1—O2	15.5 (2)
O3—C18—O4—C19	10.8 (9)	C2—C1—S1—O2	−165.07 (19)
O4A—C18—O4—C19	−82.3 (14)	C6—C1—S1—N1	−97.1 (2)
C17—C18—O4—C19	−179.6 (6)	C2—C1—S1—N1	82.3 (2)

### Hydrogen-bond geometry (Å, º)

Cg2 is the centroid of C1–C6 ring.

**Table:**

*D*—H···*A*	*D*—H	H···*A*	*D*···*A*	*D*—H···*A*
C13—H13···O1	0.93	2.44	3.012 (4)	120
C12—H12···*Cg*2^i^	0.93	2.80	3.561 (4)	140

Symmetry code: (i) *x*−1/2, −*y*−1/2, *z*−3/2.
