# Supplementary figures and images for: Crystal structure of (E)-diethyl 2-[(1-phenyl­sulfonyl-1H-indol-3-yl)methyl­idene]succinate
Source: Acta Crystallogr E Crystallogr Commun. 2015 Dec 9;71(Pt 12):o1029–30. doi: 10.1107/S2056989015023075 (PMC4719962; doi:10.1107/S2056989015023075)

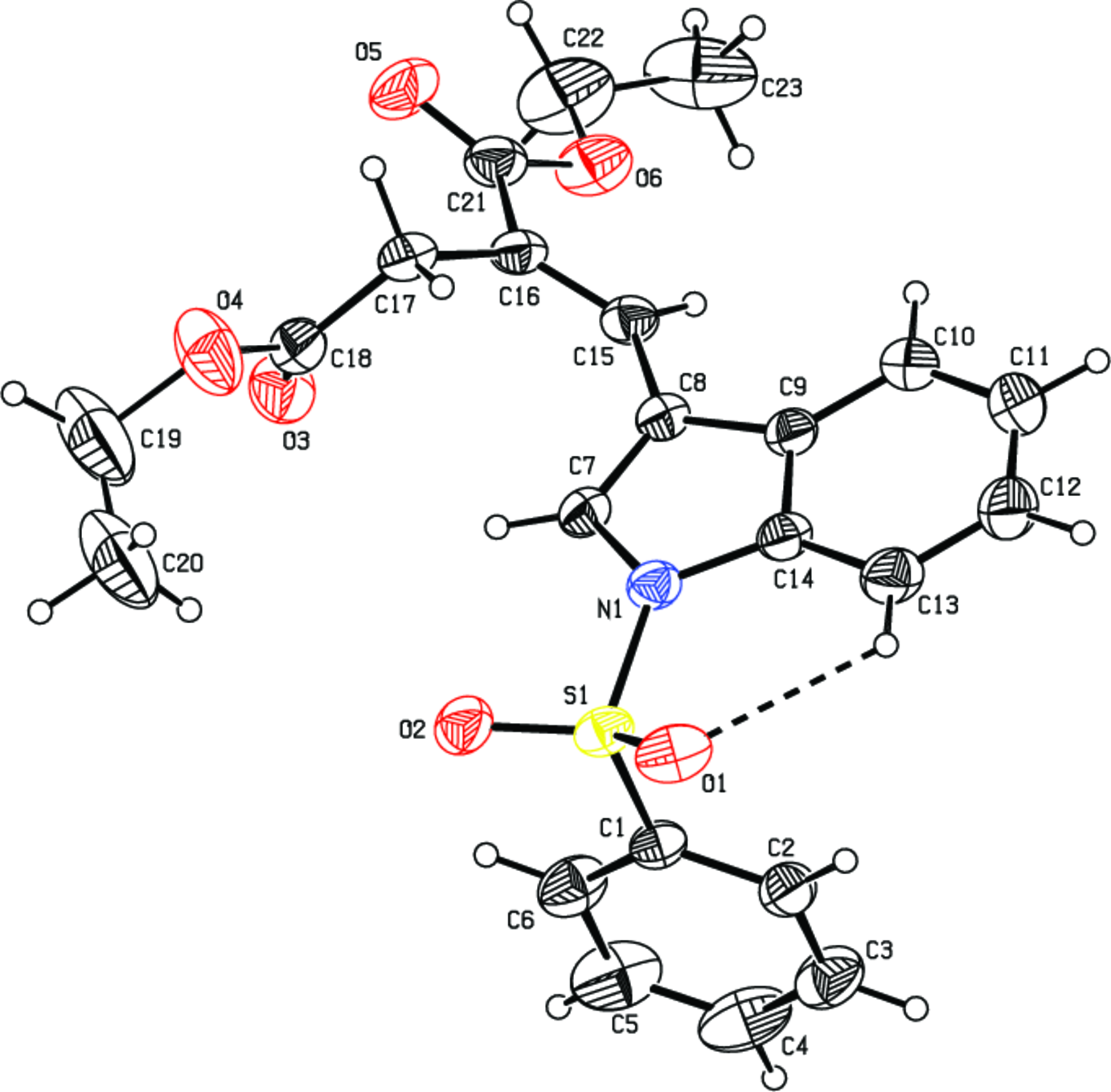

Supplement: Supplementary file 4 [file e-71-o1029-fig1.tif]
